# Effective features of e-cigarette prevention videos: A qualitative study with primary school students

**DOI:** 10.18332/tid/205839

**Published:** 2025-07-01

**Authors:** Yu Chen, Jinhui Chen, Ziliang Wang, Xinjie Zhao, Rong Gao, Yujiang Cai, Shiyu Liu, Jing Xu, Si Chen

**Affiliations:** 1 School of Art and Communication, Fujian Polytechnic Normal University, Fuzhou, China; 2 Fujian Provincial Center for Disease Control and Prevention, Fuzhou, China; 3 School of Public Health, Peking University Health Science Center, Beijing, China; 4 School of Journalism and Communication, Peking University, Beijing, China; 5 School of International Studies, Peking University, Beijing, China; 6 School of Public Health, Xi'an Jiaotong University, Xi'an, China; 7 China National Center for Food Safety Risk Assessment, Beijing, China

**Keywords:** e-cigarette prevention, child health, focus groups, health communication

## Abstract

**INTRODUCTION:**

While video-based prevention campaigns show promise in addressing the rising e-cigarette use among youth, little is known about what makes such videos effective from children’s perspectives, particularly in China’s cultural context. This study aimed to investigate which video content elements could be effective in preventing e-cigarette use among children, providing evidence-based guidance for developing e-cigarette prevention materials and information design targeting children.

**METHODS:**

Using purposive sampling, we conducted four gender-stratified focus groups with 35 primary school students (aged 12–13 years) in Beijing and Yunnan. Participants watched four stimulus videos with different messaging approaches and discussed their perceptions of effective and ineffective features. Focus group discussions were audio-recorded, transcribed, and analyzed using thematic analysis with NVivo software.

**RESULTS:**

Children identified several key features that enhanced prevention effectiveness –most prominently, explicit specific health consequences and testimonials were mentioned by a majority of participants – along with chemical component, maternal love, and emotional appeals (particularly fear). Visual elements like dynamic animations and medical expert appearances strengthened message credibility. Ineffective features included overly complex explanations, perceived inauthenticity, and news-style reporting. Notably, while some participants reported discomfort with fear-based appeals, they demonstrated high recall of these message elements.

**CONCLUSIONS:**

This study provides novel insights into developing appropriate e-cigarette prevention videos for children. Findings suggest that effective videos should combine authentic testimonials with clear health risk information, appropriate fear appeals, and engaging visual elements while avoiding overly complex or news-style presentations. These insights can guide the development of more effective youth-focused e-cigarette prevention communication strategies.

## INTRODUCTION

Electronic cigarettes (e-cigarettes), commonly known as electronic nicotine delivery systems (ENDS) and electronic non-nicotine delivery systems (ENNDS), are devices that heat liquid to produce an aerosol that users inhale^1^. These devices may or may not contain nicotine. By volume, the primary components of the solution are propylene glycol, with or without glycerin, and flavoring agents^1,2^.

E-cigarettes have experienced rapid growth through advertising, marketing, and promotional activities via internet and social media channels^2-4^. Adolescents and young adults represent the primary demographic of people who use these products, showing an alarming upward trend^5^.

Young people constitute the majority of people who use e-cigarettes, with a usage rate of 1.5% among the age group of 15–24 years. The 2018 China Adult Tobacco Survey results indicate an increase in electronic cigarette use compared to 2015, with lifetime use rising from 3.1% to 5.0% and current use increasing from 0.5% to 0.9%^6^. Electronic cigarettes have gained popularity among Chinese youth and young adults. The usage rate among Chinese young adults aged 18–29 years increased significantly from 2.0% in 2015–2016 to 2.7% in 2018–2019^7^. According to a China CDC study, 14.9% of Chinese middle school students have tried e-cigarettes, with a current usage rate of 3.0%. Survey data from 2023 indicate that the current usage rate among Chinese middle school students is 2.4%, representing a substantial population^8^. While e-cigarettes do not contain tobacco, they are harmful to health and unsafe. The use of e-cigarettes is particularly dangerous for children and adolescents. Nicotine is highly addictive, and the brain continues developing until approximately age 25 years. Children who use or even try electronic nicotine delivery systems more than double their likelihood of using traditional cigarettes later in life^1^. The World Health Organization recommends strict regulation of e-cigarettes to maximize protection of public health, including interventions to prevent non-smokers, minors, and vulnerable populations from initiating electronic cigarette use, and protecting people who do not use these products from exposure to electronic cigarette emissions^2^.

Substantial evidence now demonstrates that anti-smoking advertising campaigns can effectively prevent youth smoking and reduce smoking rates among adolescents, and similar approaches may be applicable to electronic cigarette prevention among youth^9^. An evaluation study of electronic cigarette risk communication activities conducted in Virginia confirmed the effectiveness of anti-electronic-cigarette campaigns in changing adolescent beliefs^10^. An experimental study of The Real Cost electronic cigarette prevention advertisements demonstrated consistency between perceived and actual effectiveness, showing positive outcomes in increasing risk beliefs and reducing attitudes and intentions toward electronic cigarette use^11^.

While anti-electronic-cigarette campaigns have been launched in several countries in recent years, research on developing electronic cigarette prevention advertisements and messages targeting adolescents remains limited. Message pretesting is essential for developing effective health education campaigns^12^. Most studies use quantitative methods to identify knowledge and attitude gaps; however, quantitative testing is often limited to predetermined topics identified by researchers and does not generate nuanced feedback, such as suggestions or explanations for new materials, which can be obtained through qualitative research^13^. Additionally, qualitative research can gather target audience opinions to tailor health messages for minors and inform future prevention intervention research. Minors are a key population for tobacco control because once they try smoking, addiction can easily develop and become difficult to quit.

This study is part of formative research to develop electronic cigarette prevention education campaigns targeting children, aiming to develop effective prevention education campaign materials to prevent electronic cigarette use and protect health.

## METHODS

### Sampling and data collection

We employed purposive sampling for this study. The research was conducted between November 2020 and April 2021 at two primary schools in Kunming and Beijing, with participant recruitment facilitated through school cooperation. Considering students’ expressive and comprehension abilities, we recruited only sixth-grade students from the senior primary division. Participants were stratified by gender into male and female groups, forming four groups of 6–10 students each. These two cities were selected to maximize sample variation within accessible regions. Beijing, located in northern China, has comprehensive smoke-free regulations and relatively high public awareness of tobacco control. Kunming, the capital of Yunnan Province in southern China, is situated in a major tobacco producing and consuming province with high economic dependence on tobacco, no smoke-free legislation, and comparatively lower public awareness of tobacco control.

Semi-structured group interviews were conducted using an interview guide and moderator manual developed based on research objectives and pilot-tested before data collection. The research team and moderators underwent research training, with moderators being university students who thoroughly understood the project’s methodology and objectives, and were well-versed in research procedures and techniques. Considering the participants’ age, female moderators were selected to facilitate the focus groups, aiming to create a more comfortable environment where participants could share their views openly.

Given that the research subjects were minors, informed consent was obtained from both the participants and their parents before the focus groups commenced, and discussion confidentiality was assured. Each focus group session lasted approximately 40–60 minutes and was audio-recorded. Field notes and data collection were conducted simultaneously to ensure data credibility and trustworthiness. The focus group interviews were transcribed, organized, and analyzed using NVivo software for data management.

Four international e-cigarette prevention videos of varying lengths and content, including public service announcements (PSAs) and news reports, were selected as stimulus materials for the study (Supplementary file Table 1)^14^. The videos were selected to represent a range of prevention messaging strategies (e.g. a testimonial, a chemical information video, etc.), based on prior research and consultation with tobacco control experts. All materials were sourced online and translated into Chinese with voice-overs that closely matched the original audio. International videos were chosen due to the absence of suitable Chinese-language videos at the time. Videos were selected over print materials due to their inherent characteristics: videos integrate visuals, sound effects, and color elements, making them more intuitive than images and text. Furthermore, in the era of new media, audiences predominantly access information through short videos, video websites, and social media platforms, making video content both aligned with current audience viewing preferences and the primary content format for new media platforms.

Focus group participants completed brief personal information forms before the formal interviews to verify their eligibility for the study. Any participants found ineligible would have been politely excluded and replaced with alternates, though no such cases were encountered. Following brief self-introductions guided by the moderator, participants collectively viewed the four e-cigarette prevention videos, with each video shown twice. Videos were presented in a counter-balanced order across groups to minimize order bias. Each focus group watched all four videos twice to help participants recall details and provide more considered feedback. No strong order effects were noted in the responses. The discussions then proceeded sequentially through several key areas: 1) initial reactions and feelings after watching all four stimulus videos; 2) effective elements and their reasons (what are effective and why); and 3) ineffective elements or shortcomings and their reasons (what are ineffective and why). The second and third aspects were the study’s primary focus, with moderators repeatedly explaining the meaning of ‘effective’ and ‘ineffective’ to participants during each interview. ‘Effective’ was defined as elements that would deter e-cigarette initiation, indicating positive behavioral potential, and vice versa. Moderators explored participants’ reasoning for identifying elements as ‘effective’. Subsequently, moderators further inquired about which videos or aspects participants found ineffective or deficient, investigating the underlying reasons.

### Ethics review

This study received ethical approval from the Institutional Review Board of Peking University (PU IRB), with the review number IRB00001052-20056.

### Participants

The recruitment criteria for study participants included four requirements: 1) enrollment in sixth grade at an elementary school in Kunming or Beijing; 2) aged between 12–13 years; 3) never use e-cigarettes, even a single puff; and 4) consent from teachers, students, and parents, with parents required to sign an informed consent form. The specific participant demographics are shown in Table 1.

**Table 1:** Characteristics of participants

*Characteristics*	*Categories*	*Overall* *n (%)*	*Beijing* *n (%)*	*Kunming* *n (%)*
**Gender**	Male	16 (45.7)	9 (45.0)	7 (46.7)
	Female	19 (54.3)	11 (55.0)	8 (53.3)
**Media exposure and usage**	Television	30 (85.7)	17 (85.0)	13 (86.7)
	Radio	6 (17.1)	6 (30.0)	0 (0)
	Newspaper	1 (2.9)	0 (0.0)	1 (6.7)
	Subway/Bus mobile TV	17 (48.6)	7 (35.0)	10 (66.7)
	School publicity platform	13 (37.1)	5 (25.0)	8 (55.3)
	Internet	29 (82.9)	19 (95.0)	10 (66.7)
	Other	0 (0)	0 (0)	0 (0)

### Data analysis

We employed a combined inductive and deductive approach for thematic analysis. Two researchers independently coded all transcripts and then met regularly to compare results, discuss discrepancies, and refine the coding framework. A senior researcher audited the coding process to enhance validity. We then synthesized key findings with representative quotations selected based on their relevance to the analysis objectives.

## RESULTS

The study included 35 sixth-grade elementary school students aged 12–13 years. None of the participants used e-cigarettes. Their primary media habits included television (85.7%), internet (82.9%), public transit mobile television (48.6%), and school information platforms (37.1%).

Our analysis identified several prominent themes, subthemes, and sub-subthemes. Drawing from previous advertising development and communication effectiveness evaluation dimensions, we categorized effective e-cigarette prevention video characteristics and ineffective features into two aspects: ‘theme and message features’ and ‘video characteristics and style’. The effectiveness was determined by behavioral potential, specifically ‘whether it could prevent participants from initiating e-cigarette use’ (Table 2).

**Table 2:** Thematic descriptions

*Theme*	*Subtheme*	*Sub-subtheme*
**Effectiveness and reasons**	Theme and message features	Personal testimonials
		Physician narratives
		Specific demonstration of health hazards and health risks
		Chemical components
		Maternal love and family affection
		Negative emotions
	Video characteristics and style	Vivid and engaging animation
		Fear appeal
		Metaphors
**Ineffectiveness and reasons**	Theme and message features	Unclear expression
		Unrealistic
		Irrelevant
		Negative emotions
	Video characteristics and style	Unattractive visuals
		Animation
		Monotonous format

### Effective features and reasons

Our thematic analysis revealed how participants evaluated video effectiveness across all focus groups. Despite geographical and gender differences, there was remarkable consistency in participants’ identification of key effective elements. Participants consistently identified several key features that enhanced the perceived effectiveness of e-cigarette prevention videos. Personal testimonials emerged as the most powerful element across all groups, with participants frequently citing their authenticity and credibility as particularly compelling. Health risk demonstrations and fear appeals, while sometimes creating discomfort, were recognized as effective deterrents by participants from both cities and across gender groups.

*Theme and message features*

Use of personal testimonials

Participants consistently identified real-life testimonials in the videos as effective deterrents. The reasons cited included specific medical conditions, severe consequences, authenticity, credibility, and persuasiveness. They frequently commented that testimonials showing ‘it really happened to someone’ were more believable than general health advice:

*‘Because it’s a real case ... The first impression gives you an undeniable sense of authenticity.’* (student 4, male, Beijing)

*‘It’s a real case.’* (student 1, female, Beijing)

*‘The fourth one used a real example to warn us against smoking, which was quite effective. It showed us the consequences of smoking and what could happen.’* (student 3, male, Kunming)

*‘Adam’s e-cigarette use, even though he recovered, still left him with irreversible consequences.’* (student 4, male, Kunming)

*‘In this advertisement, if Adam hadn’t fallen for that company’s pyramid scheme, he wouldn’t have used e-cigarettes and ended up in this situation.’* (student 1, male, Kunming)

*‘These real experiences show us the dangers of e-cigarettes.’* (student 2, male, Kunming)

*‘Because Adam was 18 years old when he was hospitalized for smoking, his mother was extremely worried. Initially, his mother thought the illness wasn’t serious, but later learned how severe it was. That kind of worry and anxiety, showing the family’s perspective, was quite effective.’* (student 2, female, Kunming)

Participants’ responses reflected concrete thinking patterns typical of this age group, focusing on visible, tangible evidence rather than statistical or abstract health concepts. Many explaining that testimonials were effective because ‘you can see it really happened’ or ‘the person looks really sick’.

Physician narratives

Participants identified physician involvement in video narratives as an effective feature. Participants’ respect for medical authority was evident in their responses. This reflected their developmental understanding of professional roles and authority figures:

*‘Making parents, family, and friends worry, with each doctor explaining the dangers of smoking.’* (student 1, female, Kunming)

*‘Because the doctor explained that smoking not only leads to psychological and physical depression but also causes physical diseases like pneumonia and brain damage.’* (student 2, female, Beijing)

Demonstration of specific health risks and consequences

Participants from both Beijing and Kunming agreed that specific, detailed, and visual demonstrations of e-cigarette use consequences were effective. They frequently used descriptive language such as ‘you can see what happens to your body’ or ‘it shows the bad stuff inside’, demonstrating their need for tangible evidence rather than theoretical health risks:

*‘The described health risks and the depressing tone made us better understand the severity.’* (student 4, female, Beijing)

*‘It feels like smoking has a very destructive effect on your body.’* (student 9, male, Kunming)

*‘The dangers to people were clearly explained to us.’* (student 4, female, Kunming)

*‘It controls the brain, which means it controls the entire person. Basically, the most important part is damaged.’* (student 2, female, Beijing)

*‘Making parents, family, and friends worry, with doctors explaining the dangers of smoking.’* (student 1, female, Kunming)

Chemical components

Both male and female participants reported the effectiveness of information about e-cigarettes’ chemical components and harmful substances. Participants’ interest in chemical information reflected their curiosity about ‘what’s inside’ products, a characteristic inquiry pattern at this developmental stage. Their language was concrete and often comparative, relating unknown chemicals to familiar dangerous substances:

*‘When they talked about chemical elements, I was surprised there were so many.’* (student 5, female, Beijing)

*‘They listed out all these chemical elements well, and we know they’re not good things, so they don’t need to list out specific diseases.’* (student 7, female, Beijing)

*‘I think one good aspect is that it lists out things like carbon dioxide and nicotine in e-cigarettes, and there’s a visual scene where you can see many words showing how each substance can harm your body.’* (student 3, male, Kunming)

*‘But it also explained the components of e-cigarettes, and once you know the components, you understand they're harmful.’* (student 2, female, Kunming)

Maternal love and family bonds

Several participants expressed strong identification with, resonance with, and emotional response to depictions of family relationships and maternal love. Participants’ strong response to family-centered content reflected their developmental dependence on family relationships and their emerging understanding of how their actions affect others. Participants frequently used family-referenced language such as ‘my mom would be sad’ or ‘I don't want to make my parents worry’, demonstrating how they process health risks through the lens of family relationships rather than individual consequences alone:

*‘His mother was extremely anxious. She initially thought the illness wasn’t serious, but when she learned how severe it was, she became very worried and anxious. Including family members' perspectives works well. After all, our bodies are given by our parents, so we can’t just think about ourselves.’* (student 2, female, Kunming)

*‘When we get sick, my mother is even more worried than I am, asking if I’m in pain, where it hurts, if I need her to buy anything. That’s what good mothers do. Since she’s a doctor, she knows how serious my illness might be and gets very concerned. I feel bad too. When I’m fine, my mother is happy, because not making our mothers sad is something we children should do, right?’* (student 3, female, Kunming)

Negative emotions

Participants frequently mentioned ‘disgust’ as a negative emotion, yet they considered it effective in preventing e-cigarette use. This created an interesting paradox where participants simultaneously expressed discomfort with certain content while acknowledging its effectiveness. Many participants noted that while they found some images or scenarios unpleasant, these elements were also the most memorable and likely to influence their behavior:

*‘It looks very disgusting.’* (student 1, female, Beijing)

*‘The first one is really disgusting.’* (student 3, female, Beijing)

*‘This might be disgusting, but because it’s so disgusting, it has a significant impact on people.’* (student 4, female, Beijing)

*Video characteristics and style*

Vivid and engaging animation

Participants indicated that strong visual impact and imagery were more engaging, with lively, suspenseful, and animated formats being more effective:

*‘I simply like the animated characters; they’re more interesting.’* (student 4, female, Beijing)

*‘It's dynamic.’* (student 4, male, Beijing)

*‘I think it's like a virus spreading and infecting others. I think people who come into contact with smokers might want to smoke too. I find this very useful; I'd give it 4.8 points.’* (student 5, male, Beijing)

*‘This is how the brain looks, this is how part of its structure appears.’* (student 1, female, Kunming)

*‘I think these neural and brain structures look really good.’* (student 2, female, Kunming)

*‘I think the first one is good because they use imagery that helps us imagine the many changes that happen to our body after inhaling smoke.’* (student 4, female, Kunming)

Fear appeals

Most participants reported that frightening information and imagery left a deep impression and considered it effective in preventing e-cigarette use. Despite expressing discomfort with fear-based content, participants frequently demonstrated detailed recall of frightening elements, suggesting that emotional impact enhanced memory retention:

*‘I find it particularly striking, and that thing feels particularly frightening, which is better.’* (student 4, male, Beijing)

*‘The first one feels disgusting and scary.’* (student 1, male, Beijing)

*‘I would probably quit smoking because it scared me.’* (student 5, male, Beijing)

*‘This has more impactful imagery; Adam's appearance in the hospital makes me feel afraid.’* (student 2, male, Kunming)

*‘Why the first one is good is because it feels very scary, and those bugs crawling into your brain also feel very frightening, and then it tells you this thing is not good, and those people who see it certainly don’t want bugs crawling into their brains to control them.’* (student 1, female, Kunming)

*‘They talked about many specific harms to the body, and it was particularly scary, affecting them psychologically.’* (student 4, female, Kunming)

*‘It's like a disease, a plague, and then they say it’s actually e-cigarettes. It shows blood vessels pulsing on people's faces, which makes me feel really creepy, so I think it’s quite good.’* (student 2, female, Beijing)

Metaphors

Some participants found the metaphor of ‘bugs’ particularly effective. Metaphorical representations appeared to resonate strongly with the participants, who often elaborated on the symbolic meaning of various metaphors. The use of visual metaphors seemed to help participants understand abstract concepts about addiction and health impacts in more concrete terms:

*‘It started with a bug, and as they accumulate, you’re done for.’* (student 2, female, Kunming)

*‘I think the bug is like a little black fatty. Why is it a little black fatty? Because it’s black and it’s crawled into the body.’* (student 3, female, Kunming)

*‘And there's another bug going into the lungs that's really disgusting.’* (student 3, female, Beijing)

### Ineffective features and reasons

Using the same analytical framework as above, while some features exhibited characteristics opposite to the ‘effective features’, several novel findings also emerged. Participants’ critiques were often direct and intuitive, using evaluative language such as ‘boring’, ‘fake’, or ‘too hard to understand’. Unlike adult audiences, participants’ negative evaluations were typically immediate and visceral, reflecting their limited tolerance for content that failed to engage them quickly or authentically.

*Theme and message features*

Unclear expression

Participants reported that some information presentations were too complex to comprehend and lacked clarity in their messaging. This was universally problematic across all groups, suggesting that message clarity is fundamental for effective communication with this age group. Participants noted that when they could not understand the content, they quickly lost interest and were unlikely to remember the prevention message:

*‘What exactly is happening in the brain? The discussion about brain cells and neurons didn’t address the core issues.’* (student 2, female, Beijing)

*‘People who are naturally less intelligent might also exhibit these symptoms, so it makes the harmful effects seem less significant.’* (student 4, male, Kunming)

*‘Since they mentioned it changes the brain, and some people are born with lower intelligence, it might seem like the same effect.’* (student 5, male, Kunming)

Unrealistic

Participants indicated that some content appeared exaggerated and implausible, diminishing its effectiveness. When content seemed fake or overly dramatized, participants questioned the credibility of the entire message, undermining the prevention goals:

*‘Without authentic effects, it doesn't genuinely convince people to quit smoking; these electronic versions don’t seem as serious.’* (student 2, male, Beijing)

*‘It's not realistic. Those worms are actual parasites.’* (student 5, male, Beijing)

Irrelevance

Low perceived effectiveness primarily manifested in participants’ inability to remember content, lack of relatability, and failure to comprehend consequences. Participants’ assessment of relevance was immediate and personal, often expressed as ‘that’s not about me’ or ‘I don't do that’. Their responses demonstrated that participants at this age require clear connections between health messages and their current lives:

*‘E-cigarettes still feel somewhat distant from our lives, so it’s difficult to grasp their dangerous nature.’* (student 1, female, Kunming)

Negative emotions

Some participants identified content that evoked negative emotions, such as discomfort and fear, as a drawback, suggesting they would skip such content. However, these same participants often could describe the uncomfortable details vividly:

*‘I find it uncomfortable.’* (student 1, male, Kunming)

*‘Those scenes make you feel nauseated.’* (student 2, male, Kunming)

*Video characteristics and style*

Participants identified several ineffective features, including overly childish presentation, news-like delivery, and unappealing visuals.

Visual elements

Participants expressed higher expectations for visual aesthetics. Poor visual quality was criticized across groups, with participants noting that low-quality production values made content seem less credible and professional.

*‘I deducted 0.2 points for aesthetics because some close-up shots weren’t visually appealing.’* (student 5, male, Beijing)

Animation

Participants noted that animated formats created a disconnect from real-life experiences, leading to decreased engagement. Participants’ rejection of overly simplistic animation reflected their developmental stage between childhood and adolescence. Participants particularly criticized ‘childish’ animations, using language that demonstrated their awareness of age-appropriate content and their desire to be treated as more mature audiences. Their responses indicated sensitivity to being addressed through infantile visual styles:

*‘It's purely animation-style with text and visuals.’* (student 8, female, Kunming)

*‘Animation might be more suitable for first to fourth-grade children.’* (student 5, female, Kunming)

Monotonous format

Participants criticized some videos as tedious and overly formal, resembling official news broadcasts. The news-style format was universally rejected across all groups, with participants noting that this presentation style reminded them of boring school announcements or formal health education they were required to watch rather than content they would choose to engage with voluntarily:

*‘It's too official.’* (student 1, female, Beijing)

*‘It's like a news report.’* (student 2, male, Beijing)

*‘It appears to be translated from news content.’* (student 4, male, Beijing)

## DISCUSSION

Our study investigated what kind of videos may be more likely to be effective in e-cigarette use prevention among primary school students. From thematic and informational perspectives, effective features included the use of real-life testimonials, demonstration of specific health risks, disclosure of harmful chemical constituents, maternal love, and negative emotions. Positive perceived effectiveness (PE) indicators included videos and information that provided new knowledge, prompted reflection, maintained relevance, and demonstrated persuasiveness. In testing public service announcements for smoking and e-cigarette prevention, perceived effectiveness is frequently used to evaluate advertisement or script effectiveness, as PE represents an assessment of information’s potential to influence behavioral antecedents or behaviors themselves^15,16^. Our participants’ feedback on positive PE aligns with previous research findings^15,17^. Both male and female participants emphasized the effectiveness of highlighting e-cigarette chemical components and harmful substances. Previous studies have reported similar findings, noting that mentioning chemicals in e-cigarette prevention messages creates negative associations among target audiences, leading to desired outcomes^10,13^. Additionally, participants reported that uncomfortable information effectively prevented e-cigarette use. While ‘discomfort’ represents a negative emotion, its effectiveness in preventing adverse behaviors has been documented in several studies on smoking prevention and cessation messaging^18^.

Regarding video style and characteristics, participants consistently indicated that strong visual impact and imagery were more engaging, with vivid animation formats proving most effective. Similar findings emerged from qualitative research on youth e-cigarette education initiatives across nine US states^12^. Our study also received positive feedback regarding fear appeals. Fear appeals have been extensively studied across various health communication topics, including tobacco control, with demonstrated effectiveness in behavior modification across different populations. When properly employed, fear can serve as a powerful catalyst for behavioral change. ‘All top-performing advertisements featured strong, emotionally evocative content’^19^.

Our research also identified ineffective video characteristics to avoid in future video and communication campaigns. Thematically, content should avoid vague messaging, unrealistic and irrelevant information, and news-style reporting. While participants initially identified negative emotional content like ‘discomfort’ and ‘fear’ as undesirable, suggesting they would avoid such content, they could often recall detailed aspects of these uncomfortable or frightening scenes. This finding resonates with fear appeal theories: fear-based messages can be effective in prevention only under certain conditions (e.g. when paired with efficacy information). Excessive fear may also provoke defensive responses, especially in younger audiences^20^. Our participants’ discomfort with frightening imagery, coupled with high recall of those images, suggests that fear appeals in e-cigarette prevention must be carefully calibrated, fear appeals are most effective when the audience also receives clear solutions or self-efficacy to address the fear^21^. The true effectiveness of such ‘uncomfortable’ or ‘frightening’ content warrants further investigation in future studies.

Finally, based on participants’ personal information and discussion findings, all participants regularly used TV (85.7%), followed by internet (82.9%) Subway/Bus mobile TV (48.6%) and School publicity platform (37.1%). Evidently, as internet technology evolves, obtaining health information online has become a crucial source for primary school students, fundamentally changing children’s lifestyle patterns^22^. Future e-cigarette prevention video distribution strategies must attach importance to both traditional and new media channels to effectively reach young audiences. Our research provides valuable insights for developing effective e-cigarette prevention videos targeting children, ensuring that prevention campaigns emerge from target audience perspectives rather than public health expert assumptions, highlighting the importance of qualitative research.

### Limitations

At the time of this study, no domestically produced e-cigarette prevention videos or public service announcements targeting youth were available in China. Consequently, all four stimulus videos were sourced internationally and required translation and dubbing, which may have influenced testing effectiveness. While the selected videos featured adolescents and young adults as protagonists, our participants, aged 12–13 years, may not have identified themselves as the target audience, potentially affecting their responses. Additionally, although our study participants were non-users of e-cigarettes, we did not screen for conventional tobacco use. This distinction is important as motivations for initiating e-cigarette use may differ between smokers and non-smokers, suggesting the need for differentiated prevention strategies – an area requiring further investigation. We intentionally limited the number of stimulus videos to avoid constraining participants’ imagination and creativity, allowing more time and space for open discussion. As e-cigarette use represents an emerging public health concern, international research on youth prevention education and campaign development remains limited compared to traditional tobacco control literature. Also, participants’ responses might have been influenced by social desirability, given that parents and teachers were aware of the study. The voluntary sampling could also introduce selection bias, as students who participated may differ in important ways from those who did not. We investigated a relatively small, homogeneous sample (sixth graders in two regions of China), which limits generalizability to other age groups or cultural contexts. Furthermore, our qualitative findings are descriptive and not formally quantified, so they should be interpreted as hypothesis-generating. This pioneering study in China had few precedents to draw upon and primarily relied on established health behavior change theories for guidance. From a practical perspective, we aimed to contribute to the development of effective e-cigarette prevention videos for college students by Chinese government and public health institutions.

## CONCLUSIONS

This study provides novel insights into developing appropriate e-cigarette prevention videos for children from their own perspectives. Our findings demonstrate that effective prevention videos should prioritize authentic testimonials and clear health risk information as core messaging strategies, as these elements consistently resonated with participants across both geographical locations and gender groups. The incorporation of appropriate fear appeals emerged as a complex but potentially effective approach, with participants demonstrating high recall of frightening content despite expressing discomfort, suggesting careful calibration is needed when using emotional appeals with young audiences. Engaging visual elements, particularly dynamic animations, proved essential for capturing and maintaining children’s attention, while avoiding overly complex explanations and news-style presentations emerged as critical design principles.

These findings offer evidence-based guidance for developing more effective youth-focused e-cigarette prevention communication strategies that combine authentic storytelling with age-appropriate visual design messaging approaches.

## Supplementary Material



## Data Availability

The data supporting this research are available from the authors on reasonable request.

## References

[1] WHO report on the global tobacco epidemic, 2021: addressing new and emerging products: executive summary. World Health Organization. August 17, 2021. Accessed June 20, 2025. https://www.who.int/publications/i/item/9789240032842

[2] WHO report on the global tobacco epidemic, 2023: protect people from tobacco smoke. World Health Organization. Accessed June 20, 2025. https://iris.who.int/handle/10665/372043?search-result=true&query=2023+tobacco+report&scope=%2F&rpp=10&sort_by=score&order=desc

[3] Duderstadt KG. E-cigarettes: youth and trends in vaping. J Pediatr Health Care. 2015;29(6):555-557. doi:10.1016/j.pedhc.2015.07.008 26279099

[4] Loukas A, Li X, Wilkinson AV, Marti CN. Longitudinal examination of ENDS use among young adult college students: associations with depressive symptoms and sensation seeking. Prev Sci. 2023;24(6):1068-1077. doi:10.1007/s11121-023-01572-8 37428392 PMC11210527

[5] Ma H, Kieu TK, Ribisl KM, Noar SM. Do vaping prevention messages impact adolescents and young adults? A meta-analysis of experimental studies. Health Commun. 2023;38(8):1709-1722. doi:10.1080/10410236.2023.2185578 36882378 PMC10258164

[6] Xiao L, Di X, Nan Y, Lyu T, Jiang Y. Cigarette package warnings for adult smoking cessation - China, 2018. China CDC Wkly. 2020;2(22):394-398. doi:10.46234/ccdcw2020.101 34594665 PMC8428454

[7] Zhao Z, Zhang M, Wu J, et al. E-cigarette use among adults in China: findings from repeated cross-sectional surveys in 2015-16 and 2018-19. Lancet Public Health. 2020;5(12):e639-e649. doi:10.1016/S2468-2667(20)30145-6 33271077

[8] Lyu M, Lu W, Zou L, Xiong J, Yang J. The impact of new regulations on prevention and control of e-cigarettes on adolescents in middle schools - a city in China, 2022-2023. China CDC Wkly. 2024;6(14):289-293. doi:10.46234/ccdcw2024.056 38634103 PMC11018711

[9] Wakefield M, Durrant R, Terry-McElrath Y, et al. Appraisal of anti-smoking advertising by youth at risk for regular smoking: a comparative study in the United States, Australia, and Britain. Tob Control. 2003;12(suppl 2):ii82-ii86. doi:10.1136/tc.12.suppl_2.ii82 12878778 PMC1766098

[10] England KJ, Edwards AL, Paulson AC, Libby EP, Harrell PT, Mondejar KA. Rethink vape: development and evaluation of a risk communication campaign to prevent youth E-cigarette use. Addict Behav. 2021;113:106664. doi:10.1016/j.addbeh.2020.106664 33038677

[11] Noar SM, Rohde JA, Prentice-Dunn H, Kresovich A, Hall MG, Brewer NT. Evaluating the actual and perceived effectiveness of e-cigarette prevention advertisements among adolescents. Addict Behav. 2020;109:106473. doi:10.1016/j.addbeh.2020.106473 32521287 PMC7901805

[12] Stalgaitis CA, Jordan JW, Isaac K. Creating more effective vape education campaigns: qualitative feedback from teens in nine U.S. states. Subst Use Misuse. 2023;58(3):406-418. doi:10.1080/10826084.2023.2165411 36621518

[13] Popova L, Fairman RT, Akani B, Dixon K, Weaver SR. “Don’t do vape, bro!” A qualitative study of youth’s and parents’ reactions to e-cigarette prevention advertisements. Addict Behav. 2021;112:106565. doi:10.1016/j.addbeh.2020.106565 32795737

[14] Chen Y, Liu S, Cai Y, et al. A qualitative exploration of e-cigarette prevention advertisements’ effectiveness among college students in China. Tob Induc Dis. 2024;22(June):1-12. doi:10.18332/tid/189300 PMC11184942 38895165

[15] Noar SM, Barker J, Bell T, Yzer M. Does perceived message effectiveness predict the actual effectiveness of tobacco education messages? A systematic review and meta-analysis. Health Commun. 2020;35(2):148-157. doi:10.1080/10410236.2018.1547675 30482058 PMC6538475

[16] Noar SM, Bell T, Kelley D, Barker J, Yzer M. Perceived message effectiveness measures in tobacco education campaigns: a systematic review. Commun Methods Meas. 2018;12(4):295-313. doi:10.1080/19312458.2018.1483017 31428217 PMC6699787

[17] Zhao X, Alexander TN, Hoffman L, et al. Youth receptivity to FDA’s the real cost tobacco prevention campaign: evidence from message pretesting. J Health Commun. 2016;21(11):1153-1160. doi:10.1080/10810730.2016.1233307 27736365 PMC5101168

[18] Durkin SJ, Wakefield MA, Spittal MJ. Which types of televised anti-tobacco campaigns prompt more quitline calls from disadvantaged groups? Health Educ Res. 2011;26(6):998-1009. doi:10.1093/her/cyr048 21715652

[19] Wakefield M, Bayly M, Durkin S, et al. Smokers’ responses to television advertisements about the serious harms of tobacco use: pre-testing results from 10 low- to middle-income countries. Tob Control. 2013;22(1):24-31. doi:10.1136/tobaccocontrol-2011-050171 21994276

[20] Ferguson G, Phau I. Adolescent and young adult response to fear appeals in anti-smoking messages. Young Consum. 2013;14(2):155-166. doi:10.1108/17473611311325555

[21] Jin SS, Bu K, Chen FF, et al. Correlates of condom-use self-efficacy on the eppm-based integrated model among Chinese college students. Biomed Environ Sci. 2017;30(2):97-105. doi:10.3967/bes2017.013 28292347

[22] Yang Q, Clendennen SL, Loukas A. How does social media exposure and engagement influence college students’ use of ENDS products? A cross-lagged longitudinal study. Health Commun. 2023;38(1):31-40. doi:10.1080/10410236.2021.1930671 34058919 PMC8633171

