# Supplementary material for: Effective features of e-cigarette prevention videos: A qualitative study with primary school students
Source: Tob Induc Dis. 2025 Jul 1;23:10.18332/tid/205839. doi: 10.18332/tid/205839 (PMC12215668; doi:10.18332/tid/205839)
Supplement: Supplementary file 1 [file TID-23-89-s1.pdf]

Supplementary file

Supplementary Table 1. The four stimulus videos used in the study

#	Name	Duration	Main content
1	Whose brain is it really - Advertisement	30 s	Through narration and visuals, showing the harms of e-cigarettes: There is a plague spreading. Scientists say it changes your brain, releases dangerous chemicals like formaldehyde into your blood, exposes your lungs to chromium, causing irreparable damage. It's not a parasite, not a virus, nor an infection. It's e-cigarettes.
2	Harms of e-cigarette smoking - Advertisement	1 min 40 s	Combined with animated visuals, experts explain e-cigarette marketing tactics and health risks. In marketing, e-cigarettes are often promoted as a ‘safe alternative’ to regular tobacco, with cool designs and various flavors making them more deceptive and concealed, thus attracting teenagers. A medical doctor says e-cigarettes contain large amounts of nicotine that harm teenagers' brains. The Tobacco Endgame Project Director says quitting e-cigarettes is extremely difficult, and all e-cigarettes contain harmful ingredients like diacetyl. They urge people not to risk their health.
3	Ontario teenager suffers from e-cigarette disease - News	2 min 13 s	U.S. health officials have been trying to understand what caused e-cigarette-related illness leading to the death of 28-year-old Kyle Boyd. The teenager's mother expressed concern about her son's e-cigarette use. Dr. Karen Bosma indicates that Kyle's breathing tests still show significant lung damage, similar to long-term smokers. Dr. Matthew Stanbrook states that e-cigarettes not only cause various lung diseases but will affect this boy for life. Health Minister Patty Hajdu points out that they want to take more solid actions to protect people from both e-cigarette use and advertising.
4	Doctor issues serious warning - News	4 min 34 s	A boy who uses e-cigarettes has difficulty breathing, and his mother takes him to emergency care. Doctors discover that young Adam has lung damage from e-cigarette use. Hundreds of patients nationwide have reported e-cigarette-related illnesses. Thomas Eissenberg, Deputy Director of the Center for Tobacco Products Research, says Adam initially used nicotine vaping devices sold by e-cigarette manufacturer JUUL, inhaling vape oil, which caused his immune system to react to the oil, leading to inflammation. Adam's lawyer,

			David Nelman, states that children like Adam would never smoke or try traditional combustible cigarettes if it weren't for JUUL's marketing practices. After returning home from hospitalization, Adam continues to suffer from the effects of e-cigarette harm, experiencing significant distress.

© 2025 Chen Y. et al.
